# MRI-based long-term follow-up of indolent orbital lymphomas after curative radiotherapy: imaging remission criteria and volumetric regression kinetics

**DOI:** 10.1038/s41598-023-31941-w

**Published:** 2023-03-23

**Authors:** Christian Hoffmann, Christopher Mohr, Patricia Johansson, Anja Eckstein, Andreas Huettmann, Julia von Tresckow, Sophia Göricke, Cornelius Deuschl, Christoph Poettgen, Thomas Gauler, Nika Guberina, Sourour Moliavi, Nikolaos Bechrakis, Martin Stuschke, Maja Guberina

**Affiliations:** 1 Department of Radiotherapy, University Hospital of Essen, Hufelandstrasse 55, 45147 Essen, Germany; 2 Department of Oral and Maxillofacial Surgery, University of Duisburg-Essen, Kliniken-Essen-Mitte, Essen, Germany; 3 Institute of Cell Biology (Cancer Research), Faculty of Medicine, University of Duisburg-Essen, Essen, Germany; 4 Department of Ophthalmology, University Hospital of Essen, Essen, Germany; 5 Department of Hematology, University Hospital Essen, Essen, Germany; 6 Institute for Diagnostic and Interventional Radiology and Neuroradiology, University Hospital Essen, Essen, Germany

**Keywords:** Non-hodgkin lymphoma, Radiotherapy

## Abstract

We systematically analyzed the kinetics of tumor regression, the impact of residual lesions on disease control and the applicability of the Lugano classification in follow-up MRI of orbital non-Hodgkin lymphomas that were irradiated with photons. We retrospectively analyzed a total of 154 pre- and post-irradiation MRI datasets of 36 patients with low-grade, Ann-Arbor stage I, orbital non-Hodgkin lymphomas. Patients with restricted conjunctival involvement were excluded. Lymphoma lesions were delineated and volumetrically analyzed on T1-weighted sequences. Tumor residues were present in 91.2% of all cases during the first six months after treatment. Volumetric partial response rates (> 50% volume reduction) were 75%, 69.2%, and 50% at 12–24 months, 36–48 months and > 48 months after the end of treatment. The corresponding complete response (CR) rates according to the Lugano classification were 20%, 23.1% and 50%. During a median clinical follow-up of 37 months no significant differences in progression free survival (PFS) rates were observed between the CR and non-CR group (*p* = 0.915). A residual tumor volume below 20% of the pretreatment volume should be expected at long-term follow-up beyond one year after radiotherapy.

## Introduction

Non-Hodgkin lymphomas are among the most frequent histological subtype of primary, malignant orbital tumors. In a curative setting radiotherapy is the primary type of treatment with excellent outcomes^1–10^. Whenever orbital structures are involved the whole bony orbit is irradiated with photons. At our institute orbital MRI scans are routinely performed for staging purposes and long-term follow-up. In this context we regularly receive radiological reports that mention residual post-irradiation tumor lesions of which the clinical impact is yet unknown. A common way for response evaluation in lymphomas is the application of the Lugano classification^11^. When assessing extranodal disease manifestations the full disappearance of extralymphatic sites of disease is referred to as complete response (CR). In case of residual disease, one differentiates between partial response (PR) or stable disease according to the shrinkage degree of the cross product of largest tumor diameter and the shortest axis perpendicular diameter^11^. In orbital lymphomas high but varying rates of CR are reported after irradiation (range: 52–100%) with 5-years PFS rates ranging from 75 to 100%^1,3–7,9,12–20^. However, no standardized follow-up protocol exists and the information on the applied follow-up procedures is often sparse^1,4,12,13^. Some data exists on patients receiving routine radiological follow-up procedures, however CT scans were solely or CT and MRI scans were equally performed in these studies^5–7,14,15,17,18^. Thus, the aim of this study is to share our experiences with an MRI-based long-term follow-up of orbital lymphomas that were uniformly irradiated with photons. We were particularly interested to assess the kinetics of volumetric tumor regression as the displacing orbital tumor mass is a main cause of morbidity for patients with this rare lymphoma sublocalization. We evaluate the impact of residual post-treatment lesions on local and distant tumor control probability and test the applicability of the Lugano response classification. The results should enable the radiologist and the radiation oncologist to better understand and interpret follow-up MRI in this specific lymphoma subside.

## Methods

### Patients

We retrospectively analyzed all patients diagnosed with low-grade orbital lymphoma treated whole orbit irradiation between 2000 and 2020 at the Department for Radiooncology of the University Hospital Essen. Only patients with histologically confirmed Ann Arbor Stage I lymphomas and no further extraorbital involvement were enrolled. The initial staging procedures mandatorily included an abdominal and thoracic CT scan, a bone marrow biopsy, a cranial MRI and peripheral blood analysis. An initial PET-CT scan was performed in two patients. Only one patient refused to undergo bone marrow biopsy due to his advanced age. PET-CT scans were not used during follow-up. Patients with disease limited to the conjunctiva, typically treated with en face electrons at our institution, were excluded. All patients were discussed prior to treatment in our internal, multidisciplinary cancer conference consisting of an hematologist, a radiation oncologist, a radiologist and a pathologist. A current pre-radiotherapy and at least one post-radiotherapy MRI scan image set had to be available. In total 36 patients with 38 irradiated eyes were included. Supplement Fig. S1 illustrates the recruitment process for this study. This cohort partly overlaps with the cohort of a study that we published previously. This previous study focuses on radiological side effects and health-related quality of life of 62 irradiated (1999–2020) patients with localized orbital lymphomas. The results are briefly mentioned in the discussion^21^. The drop out of patients was mainly due to the fact that MRI follow-up was not yet common in the early 2000s. Due to the rarity of the disease a significant proportion of patients were diagnosed and referred to our center by tertiary hospitals to which the patients returned for further radiological follow-up. In some cases, there was no cooperation between the different radiological departments. The median radiological follow-up was 23 months (range: 1–161 months). During that period patients underwent multiple, consecutive MRI scans. The median clinical follow-up was 37 months (range: 1–177 months). Table 1 shows the patient characteristics. Patients with involvement of the orbit and additional involvement of the conjunctiva or the lacrimal apparatus were referred to as multilocular subsite involvement.

**Table 1:** Patient characteristics.

Characteristic	No. of patients
Age at diagnosis (years)	
Average	64
Range	38–87
Sex	
Male	22
Female	14
Number of irradiated eyes	38
Histological subtype	
MALT	36
Follicular lymphoma (grade 1–2)	2
Subsite	
Orbit	18
Multilocular	16
Lacrimal gland	4

Institutional review board approval was obtained at the University Hospital Essen to conduct this retrospective study, and informed consent was waived (Ethics Committee of the Medical Faculty of the University Duisburg-Essen, ID 21-10036-BO) as only anonymized data is used. All procedures were performed according to the Declaration of Helsinki's relevant guidelines and regulations.

### Treatment techniques

All patients were treated with non-coplanar, multibeam, 5–8 megavolt photons in three-dimensional conformal radiotherapy (n = 13 lesions) or intensity-modulated radiation (IMRT) therapy (n = 25 lesions). The clinical target volume routinely encompassed the whole orbit. Two patients received treatment of the extended tumor region. Applied doses ranged from 24 Gy (2 Gy/fraction) to 30.6 Gy (1.8 Gy/fraction) (Table 2). The median dose was 30.6 Gy.

**Table 2:** Treatment characteristics.

Characteristic	No. of patients
Radiotherapy technique	
3D—non-coplanar	13
IMRT—non-coplanar	25
Radiotherapy dose (Gy/Gy/F)	
30.6 (1.8)	24
28.8 (1.8)	7
30 (2)	3
27 (1.8)	2
25.2 (1.8)	1
24 (2)	1
Clinical target volume	
Whole orbit	36
Partial orbit	2

*IMRT* intensity modulated radiotherapy, *Gy* Gray, *Gy/F* Gray per fraction.

### MRI characteristics and delineation

All available MRI datasets for each patient were imported and rigidly registrated with the planning CT within the treatment planning software ECLIPSE (Varian). The pre- and post-therapeutic gross tumor volume was preferably delineated based on a post-contrast, fat-saturated T1 sequence. For each patient the pre-treatment tumor volume was delineated on the plane where best visible (either transverse or coronal). For better comparability the post-treatment tumor residues were delineated on the same plane as the initial, pre-treatment gross tumor volume. The manual delineation and diameter measurements were performed by the first author, and supervised by the last author. The volume and the diameters were compared with all radiologic reports.

Response assessment was performed according to the Lugano classification. The longest lesion diameter was assessed on axial T1 sequences together with the shortest perpendicular diameter. The cross product of both diameters was used for further response assessment. The absence of a lesion was referred to as complete response. A reduction of 50% or more of the cross product of the longest and perpendicular diameter was referred to as partial response. In case of a regression to a largest diameter of 1 cm or less a lesion would be counted as “not measurable”. If no increase in lesion diameter was detected the response was counted as partial response. Lesions that did not qualify for a partial response but that showed no signs of progression were referred to as stable disease^11^.

In total 154 pre- and post-radiation therapy T1 weighted images were analyzed. The slice thickness ranged between 1 and 5 mm (n_1mm_ = 19, n_2mm_ = 67, n_3mm_ = 56, n_4mm_ = 2, n_5mm_ = 10). Of these 154 scans, 144 were gadolinium enhanced and 140 were fat suppressed. The predominant pulse sequences were turbo-spin-echo (tse) (n = 73) and spin-echo (se) (n = 72) sequences. As in rare cases no tse or se sequences were available we analyzed 7 volumetric interpolated breath-hold examination gradient-echo (VIBE-GRE) and 2 spectral presaturation with inversion recovery (SPIR) datasets.

As there was no available pre-irradiation T2 dataset in three patients we analyzed a total of 151 T2-weighted pre- and post-irradiation scans. The slice thickness ranged between 2 and 7 mm (n_2mm_ = 12, n_3mm_ = 113; n_5mm_ = 11, n_6mm_ = 8, n_7mm_ = 7). Due to the retrospective nature of this study and because relevant proportion of the included patients were referred to our institution by surrounding hospitals the evaluated T2 techniques varied: TIRM = 79, STIR = 36, TSE = 29, SPIR = 3, BLADE = 2, GRE* = 1, SPACE = 1.

To detect peri-tumoral edema around definable, hyper-intense tumor lesions we examined the available T2 sequences blended with T1 contrast enhanced sequences. Sequences with high detection rates for edema were preferred such as fat suppressed TIRM or STIR sequences. Tumor intensity levels in T1 and T2 sequences were compared with the contralateral M. rectus medialis and referred to as either hypo-, hyper- or isointense. Due to its retrospective nature and the long observation period of 20 years, no standardized MRI protocol was applied throughout this study. Our institute is part of a tertiary cancer center and patients that were referred to our institution by surrounding hospitals presented with varying protocols. The most recent staging and follow-up MRI protocol with a dedicated head coil according to our standard operating procedures is depicted in Table 3.

**Table 3:** Standard gadolinium-enhanced MRI protocol for orbital lymphomas.

Modality	Plane	Slice thickness (mm)
T1 tse	Transverse	2
T2 tse	Transverse	2
T2 stir	Coronal	3
T2 FLAIR	Transverse	5
T1 tse fat saturated	Transverse	2
T1 tse fat saturated	Coronal	3
T1 mprage	Transverse	1
T1 mprage	Coronal	3
T1 mprage	Saggital	1

*tse *turbo spin echo, *stir* short tau inversion recovery, *FLAIR* fluid-attenuated inversion recovery.

### Statistical analysis

SPSS Statistics software (version 27.0, IBM) or SAS (version 14.1, SAS Institute) were used for statistical analysis. Disease control rates were calculated according to the Kaplan–Meier method from the end of treatment till the date of the last clinical follow-up or date of recurrence. We analyzed possible influencers such as disease sublocalization or initial tumor volume on the probability for a patient to achieve a CR during follow-up. For statistical purposes the data was dichotomized into two groups regarding the “overall achievement of a CR”. To do so the last follow-up scan was analyzed whether a CR was achieved or not. For determination of statistically significant differences, the unpaired Student’s t-test, the Mann–Whitney U test, the Fisher’s exact test and the Log Rank test were performed. p-values below 0.05 were regarded as statistically significant. The change of the relative tumor volume normalized to the pretreatment volume over time from the end of radiotherapy was fitted to a biexponential model with two components and offset for the final residual volume using Procedure NLlN from SAS.

### Informed consent

Institutional review board approval was obtained at the University Hospital Essen to conduct this retrospective study, and informed consent was waived (Ethics Committee of the Medical Faculty of the University Duisburg-Essen, ID 21-10036-BO) as only anonymized data is used.

## Results

### MRI features

**Figure 1:**
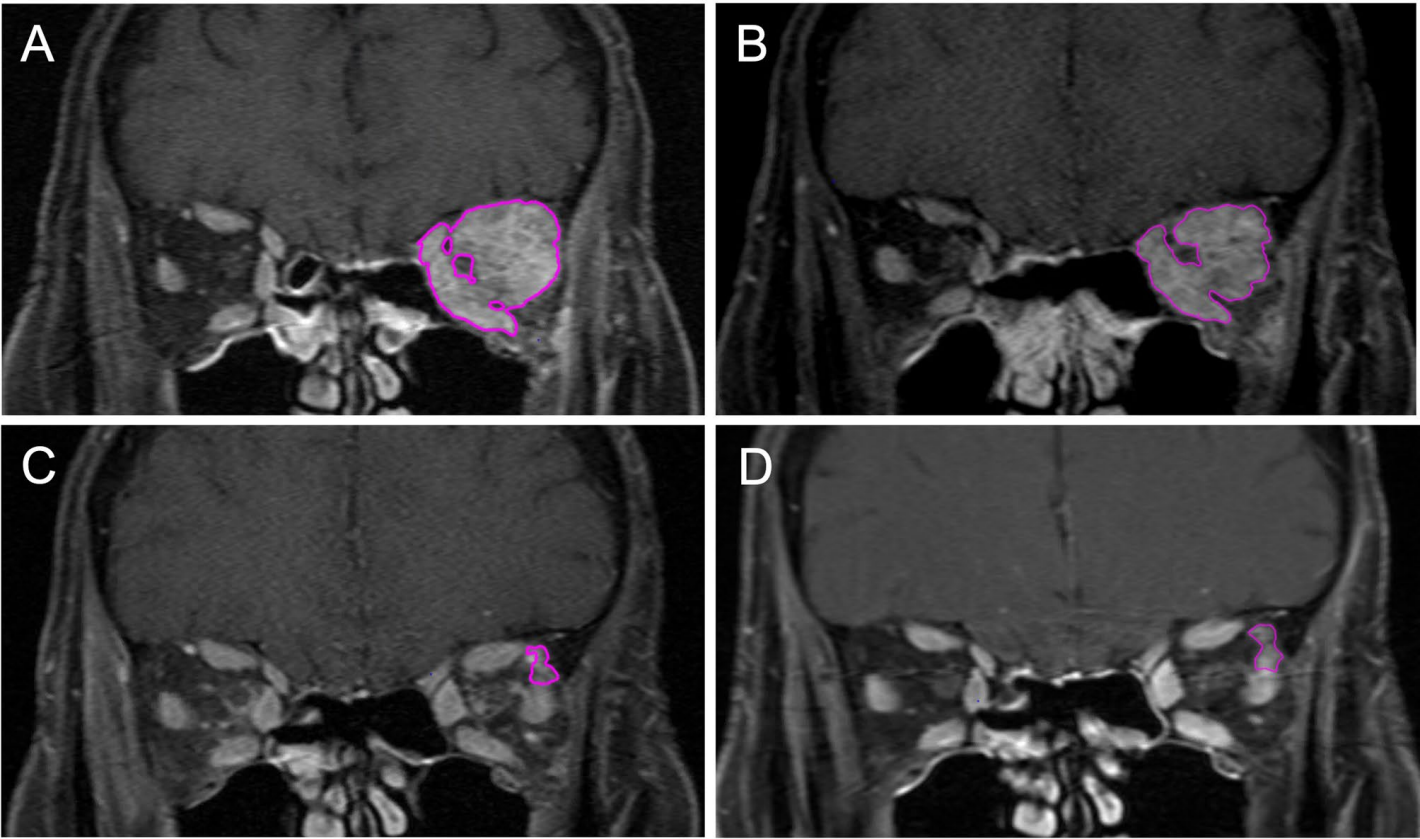
Patient with orbital MALT lymphoma before (**A**) and 2 (**B**), 15 (**C**) and 40 (**D**) months after irradiation. The well definable tumor residue is marked in magenta.

All lesions were detectable on both T1 and T2 weighted sequences before treatment. Lymphoma lesions were either iso- (76.3%) or hyperintense (23.7%) on pre-treatment T1 weighted images. Shortly after irradiation (< 6 months), residues were still detectable on T1 weighted images in 91.2% of all patients. However, at that time point in 29.4% of the cases no residue could be detected on T2 weighted images. In general, T1 weighted images showed higher detection rates for tumor residues than the T2 weighted images. We therefore decided to perform all volumetric analyses on T1-weighted images. Figure 1 shows an exemplary MRI set.

### Characterization of residual tumor volume reduction on T1 weighted images

**Figure 2:**
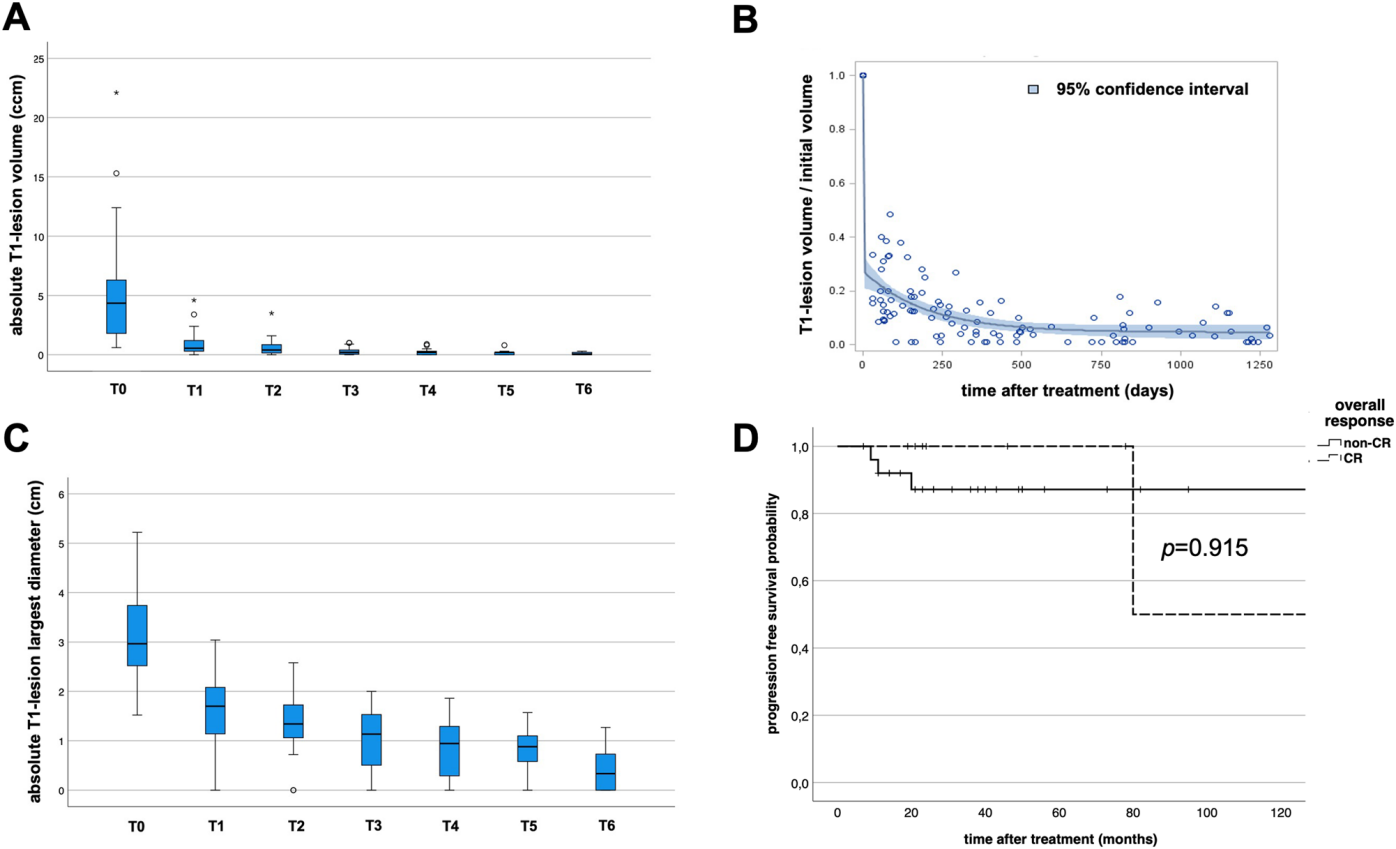
Volumetric data of orbital lymphomas prior to irradiation (T0); < 6 months (T1), 6–12 months (T2), 12–24 months (T3), 24–36 months (T4) 36–48 months (T5) and > 48 months (T6) after treatment. (**A**) The absolute tumor volume sharply decreases after treatment. (**B**) Exponential model with two components and final offset. The relative lesion volume was normalized to the pre-treatment volume. The x-axis shows the time after the last treatment fraction in days. The fast decay already occurs before the first follow-up MRI. The slow component accounts for 22.8% (95% confidence interval 16.9–28.7%) of the decay. It has a half time of 139.2 days (95% confidence interval 87.6–338.1 days). (**C**) The median, absolute lesion diameter quickly falls below 1 cm. (**D**) Kaplan–Meier plot showing the progression free survival rates of patients that achieved a complete response (dotted line) versus patients with persisting tumor residue after treatment (non-CR group, solid line). The differences were not statistically significant (Log Rank test, *p* = 0.915).

The median tumor volume on T1 weighted images before irradiation was 4.35 cm^3^ (range: 0.6–22.1 cm^3^). During the first six months after irradiation, the median tumor residue volume decreased by 83.1% (SD ± 11.3%) (Fig. 2A). During the first and second year of follow up we observed a further decrease in median residue volume. When compared to pre-treatment levels we saw a reduction to a relative volume of 11.1% (SD ± 7.8%) and 4.7% (SD ± 5.0%) respectively. Figure 2B shows a biexponential model with two components and offset for the final residual volume. The post-treatment lesion volume was normalized to the pre-treatment volume, with time being calculated from the last treatment fraction in days. The fast decay already occurred within the treatment period and accounted for 72.6% of the decay (95% confidence interval (CI) 65.7–79.5%). The slow component accounted for 22.8% of the decay (95% CI 16.9–28.7%) with a half time of 139.2 days (95% CI 87.6–338.1 days). The final relative residual volume was 4.6% (95% CI 1.7–7.5%) and was not significantly dependent on the absolute pretreatment volume.

### Response assessment according to the Lugano classification

The median largest diameter on T1 weighted images before irradiation was 2.97 cm (range: 1.52–5.22 cm). We observed the same tendency for tumor shrinkage as it was observed for the total tumor volume (Fig. 2A,C).

The clear tendency for progressive tumor shrinkage during long-term follow-up was reflected by steadily growing local CR rates. At less than 6 months, 6–12 months, 12–24 months, 24–36 months, 36–48 months and more than 48 months after treatment the corresponding CR rates were 8.8%, 8.7%, 20%, 25%, 23.1% and 50% respectively. The rate of patients with a stable disease up to 6 months after irradiation was 14.7%. All patients presented with at least a partial response after more than 6 months after treatment. None of the patients showed local disease progression. The correlation coefficient between the volumetric (> 50% volume reduction) and the Lugano partial response rate was r = 1. Four patients presented with outfield reoccurrences after 9, 11, 20 and 80 months. In two patients the new lesion was solely located in the contralateral eye. Both patients were again and successfully treated by radiation therapy. One of the contralateral eye relapses occurred after 80 months and achieved a CR in both eyes.

There was no statistically significant difference in PFS during the clinical follow-up period for the non-CR group when compared to the CR group (*p* = 0.915) (Fig. 2D).

The largest, pre-treatment tumor diameter on T1-weighted images was the only significant prognostic factor for the overall achievement of a CR (*p* = 0.011). Patients with pre-treatment lesions larger than 3 cm were significantly less likely to achieve a CR (*p* = 0.008). Neither pre-treatment geometric tumor features such as total tumor volume (*p* = 0.151) or the largest perpendicular tumor diameter (*p* = 0.194) nor radiological features such as muscle invasion (*p* = 0.556), lacrimal gland invasion (*p* = 0.510), coating of the optical nerve (*p* = 0.369) were associated with significantly altered overall CR rates.

### Influence of total dose on tumor volume reduction

In recent years a trend for dose reduction in adnexal lymphomas evolved^5,8,10,14,22–25^. We were therefore interested to find out if dose reduction below 30 Gy had an influence on the tumor remission kinetics. At our institution 30.6 Gy (1.8 Gy/fraction) was the standard treatment regime until 2015. Since then, we routinely applied 28.8 Gy (1.8 Gy/fraction) for small and 30.6 Gy (1.8 Gy/fraction) for large tumors. As shown in Table 2, 25 patients received total doses of 30 Gy or more whereas nine patients were treated with a cumulative dose of less than 30 Gy. No difference in relative or absolute tumor volume reduction or in relative or absolute largest tumor diameter reduction between both groups could be detected at any time point during follow-up.

## Discussion

Radiotherapy is the current standard treatment for localized, indolent lymphomas of the orbit and profound data exists that underlines its effectiveness^2,10,13,16,26^. In a previous study—of which the patient cohort partly overlaps with the cohort of the present study—we presented promising results regarding treatment related toxicities. In 62 patients with irradiated orbital lymphomas none of the acute toxicities was higher than CTCAE grade 1. The predominant late toxicities were dry eyes (21.2%) of CTCAE Grade < 2 and radiation induced cataracts (19.7%). However, we are routinely faced with radiological reports that mention residual lesions on post-treatment MRI and the impact of these residues on disease control is yet unknown. We therefore analyzed a total of 154 pre- and post-radiation therapy MRI datasets of 38 eyes that were uniformly treated with photon radiation therapy. All patients had Ann Arbor Stage I lymphomas without any sign of extraorbital involvement. More than 90% of all cases showed post-treatment residues during the first six months after treatment. Gadolinium enhanced, fat suppressed T1 weighted images proved to be more sensitive for the detection of residues than T2 weighted images and were therefore used for further volumetric analysis. We applied an exponential model with two components and an offset for the permanent residual volume that summarizes the relative lesion volume change. The fast decay accounted for 72.6% of the decay and already occurred during treatment and before the first follow-up MRI. The slow component accounted for 22.8% of the decay (95% CI 16.9–28.7%) with a half time of 139.2 days (95% CI 87.6–338.1 days) after the last treatment session. We furthermore applied the Lugano response classification and showed that the non-achievement of a CR was not associated with an impaired PFS (*p* = 0.915). The largest, pre-treatment tumor diameter on T1-weighted images was the only significant prognostic factor for the overall achievement of a CR (*p* = 0.011). Two patients developed isolated relapses in the contralateral eye that were detected early on follow-up MRI. Both were again and successfully irradiated which underlines the importance of thorough radiological follow-up procedures.

In irradiated orbital lymphomas high but varying rates of CR (range: 52–100%) are reported^1,4–7,12–18,20^. Due to the fact of missing follow-up standardization some authors do not provide information on the radiological follow-up procedures^4,12,13^ whereas in one publication radiological follow-up was only performed if appropriate^1^. Some data exists on patients receiving routine radiological follow-up procedures, however CT scans were solely or CT and MRI scans were equally performed in these studies^5–7,14,15,17,18,20^. Furthermore, the before mentioned studies do not differentiate between orbital and conjunctival lesion^1,4–7,12–18,20^.

From the perspective of a radiologist our data suggests that the before mentioned CR rates might—due to insensitive imaging techniques—be overestimated. This finding could be supported by previous studies that mention post-irradiation orbital lesions on MRI^27,28^. Recent studies tried to replace [^18^F]FDG-PET based response assessment by whole-body diffusion-weighted imaging MRI in juvenile Hodgkin’s lymphoma. In this context MRI was highly sensitive for the detection of residual masses but the lack of metabolic information resulted in a tendency to underestimate the true treatment response^29–31^. It remains unclear whether the observed residues contain active tumor cells or rather consist of scarred tissue. A previous study histologically examined resected, post-treatment residual masses of Hodgkin’s lymphoma and characterized them as oligocellular, hyalinized masses^32^. In contrast, only low grade lymphomas were included in this study. PET diagnostics show considerable limitations, especially in the orbital region, primarily due to the physiologic [^18^F]FDG uptake by extraocular muscles and the small volumes of the residual lesions^33^. Ultimately, [^18^F]FDG-, [^68^Ga]Pentixafor-PET or diffusion-weighted imaging MRI might be used in further follow-up studies to better characterize the observed residues^20,34–36^. However, only 80% of orbital MALT lymphomas show an initial, positive [^18^F]FDG-uptake^20,37^. Our volumetric data is in line with previously published data by Wang et al. who performed an early CT based treatment response assessment just after 20–27 Gy. The tumor decline rate at that time point was 66.4% in [^18^F]FDG-avid lesions and 62.5% in lesions without initial [^18^F]FDG-avidity^20^. Furthermore, Jung et al. reported a median tumor volume reduction on MRI in eleven irradiated eyes of approximately 90% during the first 12 months after treatment^28^. This underlines how radiotherapy generates a rapid treatment response which makes it extremely valuable in cases in which critical orbital structures are threatened by the displacing tumor mass. Up to our knowledge the first large scale study that provides and matches both long-term clinical and MRI data of irradiated orbital lymphomas.

A main limitation of this study is—due to its retrospective nature—the absence of a standardized MRI protocol. Furthermore, patients that were referred to our institution by other hospitals presented with protocols that consisted of varying acquisition techniques as well as varying slice thicknesses. There are no quality criteria regarding the slice thickness in the Lugano classification, but the RECIST 1.1 criteria suggest that the size of a measurable lesion at baseline should be two times the slice thickness^11,38^. In our cohort this was the case for all pre-treatment and for 67% of the post-treatment datasets. Furthermore, by co-registration with the initial imaging within the radiation platform additional layers can be interpolated. This might have had a negative impact on the accuracy of our volumetric analysis and even our data might overestimate the true CR rates. As the observed residues are mainly visible on T1 weighted, fat saturated sequences and the median residue diameter was 1.14 cm at 12–24 months after treatment we propose a slice thickness of 1 mm in these sequences. In some cases, repeated T2 sequences with high sensitivity for edema are useful to distinguish tumor residues from healthy tissues such as muscles and the lacrimal gland. To us, coronal sections were more helpful than transverse sections in these locations. T2 TIRM or STIR sequences proved to be highly useful in this study.

## Conclusion

The post-irradiation shrinkage of orbital, low-grade non-Hodgkin lymphomas can be adequately described by a biexponential model. Irradiation induces a significant tumor volume shrinkage which to a significant degree already occurs during treatment. A significant residual volume of 4.6% of the initial volume remains over the whole group of patients. Absolute but not relative residual tumor volume is dependent on the pretreatment tumor volume. From our clinical data it can be concluded that the discrimination of a complete remission from a non-complete-remission outcome according to the Lugano classification does not have a prognostic value. Following whole-orbit irradiation, a relative residual tumor volume below 20% of the pre-treatment volume should be expected for the individual patient.

## Supplementary Information

Supplementary Information.

## Data Availability

The datasets generated and analyzed during the current study are not publicly available due to the fact that they contain personal clinical data. They are available from the corresponding author on reasonable request in an anonymized form.

## References

[1] Fung CY (2003). Ocular adnexal lymphoma: Clinical behavior of distinct World Health Organization classification subtypes. Int. J. Radiat. Oncol. Biol. Phys..

[2] Yahalom J (2015). Modern radiation therapy for extranodal lymphomas: Field and dose guidelines from the International Lymphoma Radiation Oncology Group. Int. J. Radiat. Oncol. Biol. Phys..

[3] Parikh RR (2015). Long-term outcomes and patterns of failure in orbital lymphoma treated with primary radiotherapy. Leuk. Lymphoma.

[4] Bayraktar S, Bayraktar UD, Stefanovic A, Lossos IS (2011). Primary ocular adnexal mucosa-associated lymphoid tissue lymphoma (MALT): Single institution experience in a large cohort of patients. Br. J. Haematol..

[5] Rehn S (2020). Radiotherapy dose and volume de-escalation in ocular adnexal lymphoma. Anticancer Res..

[6] Hata M (2011). Treatment effects and sequelae of radiation therapy for orbital mucosa-associated lymphoid tissue lymphoma. Int. J. Radiat. Oncol. Biol. Phys..

[7] Nam H, Ahn YC, Kim YD, Ko Y, Kim WS (2009). Prognostic significance of anatomic subsites: Results of radiation therapy for 66 patients with localized orbital marginal zone B cell lymphoma. Radiother. Oncol..

[8] Platt S (2017). Extranodal marginal zone lymphoma of ocular adnexa: Outcomes following radiation therapy. Ocul. Oncol. Pathol..

[9] Harada K (2014). Localized ocular adnexal mucosa-associated lymphoid tissue lymphoma treated with radiation therapy: A long-term outcome in 86 patients with 104 treated eyes. Int. J. Radiat. Oncol. Biol. Phys..

[10] Olsen TG (2019). Orbital lymphoma—An International Multicenter Retrospective Study. Am. J. Ophthalmol..

[11] Cheson BD (2014). Recommendations for initial evaluation, staging, and response assessment of Hodgkin and non-Hodgkin lymphoma: The Lugano classification. J. Clin. Oncol..

[12] Desai A (2017). Long-term course of patients with primary ocular adnexal MALT lymphoma: A large single-institution cohort study. Blood.

[13] Rasmussen PK (2014). Ocular adnexal follicular lymphoma: A multicenter international study. JAMA Ophthalmol..

[14] Goda JS (2011). Localized orbital mucosa-associated lymphoma tissue lymphoma managed with primary radiation therapy: Efficacy and toxicity. Int. J. Radiat. Oncol. Biol. Phys..

[15] Suh CO (2006). Orbital marginal zone B-cell lymphoma of MALT: Radiotherapy results and clinical behavior. Int. J. Radiat. Oncol. Biol. Phys..

[16] Jeon YW (2018). Comparison of selection and long-term clinical outcomes between chemotherapy and radiotherapy as primary therapeutic modality for ocular adnexal MALT lymphoma. EClinicalMedicine.

[17] Uno T (2003). Radiotherapy for extranodal, marginal zone, B-cell lymphoma of mucosa-associated lymphoid tissue originating in the ocular adnexa: A multiinstitutional, retrospective review of 50 patients. Cancer.

[18] Son SH (2010). Primary radiation therapy in patients with localized orbital marginal zone B-cell lymphoma of mucosa-associated lymphoid tissue (MALT Lymphoma). Int. J. Radiat. Oncol. Biol. Phys..

[19] Ohga S (2013). Radiotherapy for early-stage primary ocular adnexal mucosa-associated lymphoid tissue lymphoma. Anticancer Res..

[20] Wang W (2022). The role of (18)F-FDG PET/CT in diagnosis and treatment evaluation for ocular adnexal mucosa-associated lymphoid tissue lymphoma. Br. J. Radiol..

[21] Hoffmann C (2022). Long-term follow-up and health-related quality of life among cancer survivors with stage IEA orbital-type lymphoma after external photon-beam radiotherapy: Results from a longitudinal study. Hematol. Oncol..

[22] Stafford SL (2001). Orbital lymphoma: Radiotherapy outcome and complications. Radiother. Oncol..

[23] Hoskin PJ (2014). 4 Gy versus 24 Gy radiotherapy for patients with indolent lymphoma (FORT): A randomised phase 3 non-inferiority trial. Lancet Oncol..

[24] Lee MJ, Lee MY, Choe JY, Choi SH, Kim HJ (2021). Ultra-low-dose radiation treatment for early-stage ocular adnexal MALT lymphoma. Eur. J. Ophthalmol..

[25] Lowry L (2011). Reduced dose radiotherapy for local control in non-Hodgkin lymphoma: A randomised phase III trial. Radiother. Oncol..

[26] Lee J (2019). Patterns of care for orbital marginal zone B-cell lymphoma of mucosa-associated lymphoid tissue in Korea throughout 2016: Results from a multicenter cross-sectional cohort study (KROG 16–19). Asia Pac. J. Clin. Oncol..

[27] Politi LS (2010). Ocular adnexal lymphoma: Diffusion-weighted MR imaging for differential diagnosis and therapeutic monitoring. Radiology.

[28] Jung SK (2015). Suggestion of response evaluation criteria in patients with ocular adnexal mucosa-associated lymphoid tissue lymphoma (OAML). Ann. Hematol..

[29] Spijkers S (2021). Whole-body MRI versus an [(18)F]FDG-PET/CT-based reference standard for early response assessment and restaging of paediatric Hodgkin's lymphoma: A prospective multicentre study. Eur. Radiol..

[30] Latifoltojar A (2019). Whole-body MRI for staging and interim response monitoring in paediatric and adolescent Hodgkin's lymphoma: A comparison with multi-modality reference standard including (18)F-FDG-PET-CT. Eur. Radiol..

[31] Littooij AS (2015). Whole-body MRI-DWI for assessment of residual disease after completion of therapy in lymphoma: A prospective multicenter study. J. Magn. Reson. Imaging.

[32] Chen JL, Osborne BM, Butler JJ (1987). Residual fibrous masses in treated Hodgkin's disease. Cancer.

[33] Kalemaki MS (2020). PET/CT and PET/MRI in ophthalmic oncology (Review). Int. J. Oncol..

[34] Mayerhoefer ME (2015). Evaluation of diffusion-weighted magnetic resonance imaging for follow-up and treatment response assessment of lymphoma: Results of an 18F-FDG-PET/CT-controlled prospective study in 64 patients. Clin. Cancer Res..

[35] Mayerhoefer ME (2022). CXCR4 PET/MRI for follow-up of gastric mucosa-associated lymphoid tissue lymphoma after first-line Helicobacter pylori eradication. Blood.

[36] Mayerhoefer ME (2016). Can interim 18F-FDG PET or diffusion-weighted mri predict end-of-treatment outcome in FDG-avid MALT lymphoma after rituximab-based therapy? A preliminary study in 15 patients. Clin. Nucl. Med..

[37] Park HL (2019). Role of F-18 FDG PET/CT in non-conjunctival origin ocular adnexal mucosa-associated lymphoid tissue (MALT) lymphomas. EJNMMI Res..

[38] Eisenhauer EA (2009). New response evaluation criteria in solid tumours: Revised RECIST guideline (version 1.1). Eur. J. Cancer..

