# Supplementary material for: MRI-based long-term follow-up of indolent orbital lymphomas after curative radiotherapy: imaging remission criteria and volumetric regression kinetics
Source: Sci Rep. 2023 Mar 23;13:4792. doi: 10.1038/s41598-023-31941-w (PMC10036339; doi:10.1038/s41598-023-31941-w)
Supplement: Supplementary file 1 — Supplementary Information. [file 41598_2023_31941_MOESM1_ESM.pdf]

Flow Chart

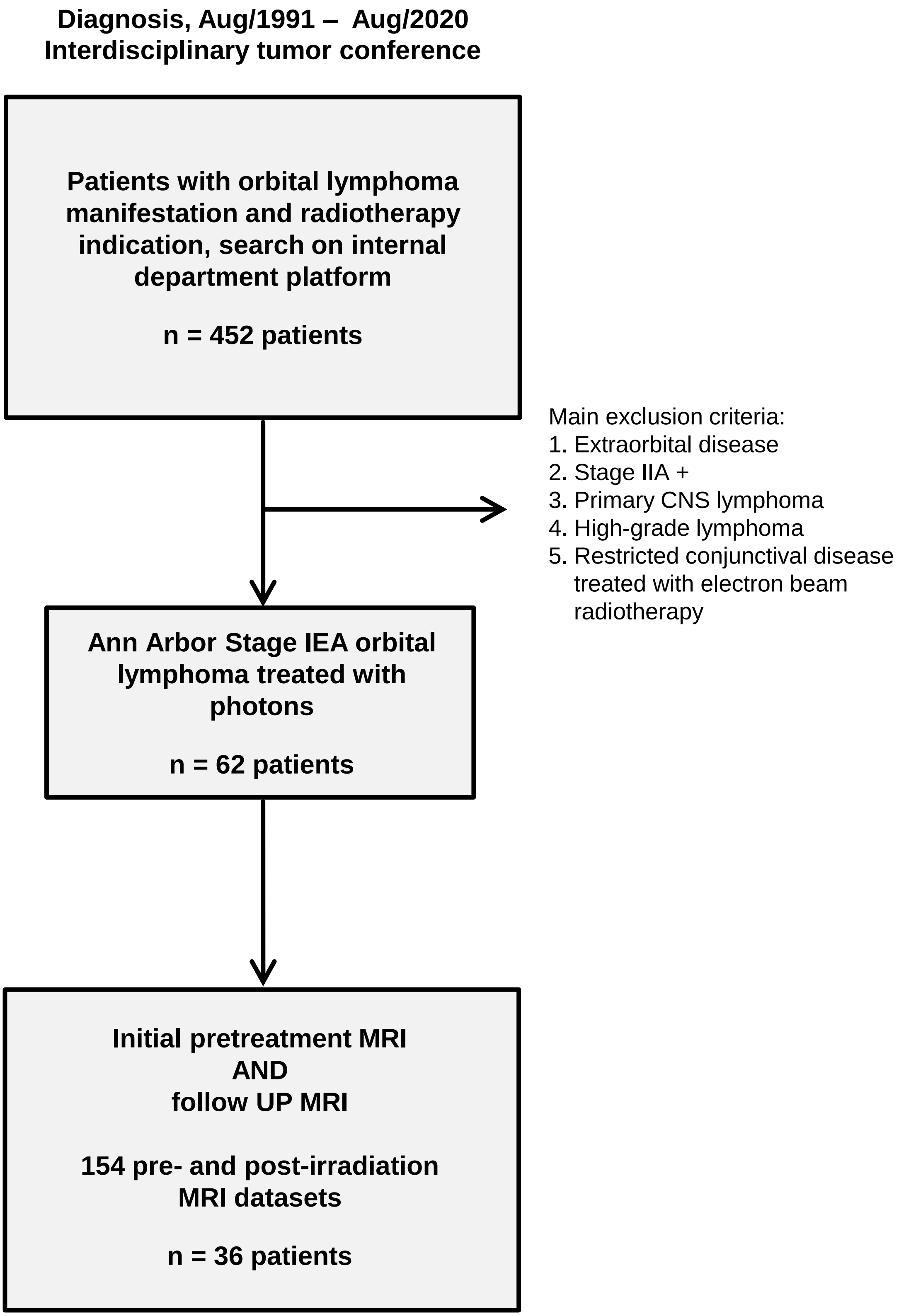

Supplement figure S1
Flow chart highlighting the main inclusion and exclusion criteria of the study
